# Use of Glucagon-Like-Peptide 1 Receptor Agonists and Risk of Fracture as Compared to Use of Other Anti-hyperglycemic Drugs

**DOI:** 10.1007/s00223-015-0037-y

**Published:** 2015-07-17

**Authors:** Johanna H. M. Driessen, Hein A. W. van Onzenoort, Jakob Starup-Linde, Ronald Henry, Andrea M. Burden, Cees Neef, Joop P. van den Bergh, Peter Vestergaard, Frank de Vries

**Affiliations:** Division of Pharmacoepidemiology and Clinical Pharmacology, Utrecht Institute of Pharmaceutical Sciences, Utrecht University, Utrecht, The Netherlands; Care and Public Health Research Institute (CAPHRI), Maastricht, The Netherlands; Department of Clinical Pharmacy and Toxicology, Maastricht University Medical Centre+, Maastricht, The Netherlands; Department of Pharmacy, Radboud University Nijmegen Medical Centre, Nijmegen, The Netherlands; Department of Clinical Medicine, Aalborg University, Aalborg, Denmark; Department of Endocrinology and Internal Medicine, Aarhus University Hospital, Aarhus, Denmark; Department of Medicine, Maastricht University Medical Centre+, Maastricht, The Netherlands; Cardiovascular Research Institute Maastricht, Maastricht University Medical Centre+, Maastricht, The Netherlands; Department of Internal Medicine, Maastricht University Medical Centre+, Maastricht, The Netherlands; Department of Internal Medicine, VieCuri Medical Centre, Venlo, The Netherlands; Department of Endocrinology, Aalborg University Hospital, Aalborg, Denmark; MRC Epidemiology Lifecourse Unit, Southampton General Hospital, Southampton, UK

**Keywords:** GLP-1 RA, Fracture, Type 2 diabetes mellitus, Case–control

## Abstract

Glucagon-like peptide-1 receptor agonists (GLP-1 RAs) are a new class of drugs that might have a potential beneficial effect on bone metabolism. Data on the effect of GLP-1 RAs and fracture risk are lacking. The aim of the present study was to investigate the association between the use of GLP-1 and the risk of fracture. A case–control study was performed using Danish National Health Service data. Cases were those who sustained a fracture and controls were those without a fracture during the study period (2007–2011), all aged 18 years and above. Conditional logistic regression estimated the odds ratios (OR) of fracture with current use of DPP4-I use. Analyses were adjusted for comorbidities and recent drug use. Among cases (*n* = 229,114), there were 6993 current non-insulin anti-diabetic drug (NIAD) users (excluding incretin users) and 255 GLP-1 RA users. Similarly, among controls (*n* = 229,114), 7209 were NIAD users (excluding incretin users) and 220 were GLP-1 RA users. Current GLP-1 RA use was not associated with a decreased risk of fracture [adjusted (adj.) OR 1.16; 95 % CI 0.83–1.63]. Osteoporotic fracture risk was also not associated with current GLP-1 RA use (adj. OR 0.78; 95 % CI 0.44–1.39). In our nation-wide case–control study, we identified that the use of GLP-1 RA was not associated with fracture risk as compared to the use of other anti-hyperglycemic drugs. Additionally, current GLP-1 RA use, stratified by cumulative or average daily dose, is not associated with fracture risk. Further research should focus on long-term use of GLP-1 RA and fracture risk.

## Introduction

The risk of fracture is significantly increased in patients with type 2 diabetes mellitus (T2DM) [1]. It has been hypothesized that a reduced bone strength or bone quality plays a role in patients with T2DM [2]. Alternatively, it has been suggested that anti-hyperglycemic drugs might affect fracture risk. For instance, observational studies showed that the use of thiazolidinediones is associated with a 1.3–1.9-fold [3, 4] increased risk of fracture, in particular in women, as compared to the use of other anti-hyperglycemic drugs. Insulin use has been associated with an elevated fracture risk [5], whereas metformin might be associated with a reduced fracture risk [6].

Glucagon-like peptide-1 receptor agonists (GLP-1 RAs), such as exenatide and liraglutide, are a new class of drugs in the treatment of T2DM. GLP-1 RAs may have a potential beneficial effect on bone metabolism, established by binding of GLP-1 RAs to a GLP-1 receptor on osteocytes and osteoblasts, as shown in in vitro studies [7–9]. Consequently, this may result in an increased bone formation and a decreased bone resorption [8, 10]. As a result of this process, we hypothesize that this then may lead to a reduced risk of fracture.

However, currently, there are limited data, particularly epidemiologic, in the literature. A recent meta-analysis of randomized clinical trials (*n* = 4255, mean duration of included studies 67.4 weeks) did not show a reduced risk of fracture with the use of GLP-1 RAs [11] as compared to the use of other anti-hyperglycemic drugs. To our knowledge, there is only one observational study examining fracture risk with GLP-1 RA use which showed no association [12]. In particular, studies with longer durations of use or stratified to daily dose are required to best understand the association between GLP-1 RA use and fracture risk. We therefore sought to examine the association between GLP-1 RA use compared to other anti-hyperglycemic drug use and fracture risk in a nation-wide case–control study.

## Methods

### Data Source

We utilized data from the Danish National Health Service, which cover all contacts to the health sector, and include approximately 5.2 million individuals in 1995 and 5.5 million in 2011 [13]. The unique 10-digit civil registry number was used to link population-based registries and generate a complete hospital discharge and prescription history for each individual [14]. Data on vital status (e.g., change of address and date of death) for the entire Danish population have been collected since 1968 in the Civil Registration System. All inpatient contacts have been registered through the Danish National Hospital Discharge Register [15] since 1977, and outpatient visits to hospitals, outpatient clinics, and emergency rooms have been included since 1995. In Denmark, prescription records are sent directly to a Register of Medicinal Product Statistics (i.e., a prescription database) at the Danish Medicines Agency. The prescription database includes information on patient’s civil registry number, the type and amount of drug prescribed according to the Anatomical Therapeutic Chemical (ATC) classification system, and the date when the prescription was filled. Pharmacy data have been collected since January 1, 1996. This study was subject to control by the National Board of Health and the Danish Data Protection Agency.

### Study Design

The study was designed as a case–control study. Cases were all subjects, both genders and aged 18 years and above, who sustained a fracture, low or high trauma, (International Classification of Diseases and Related Health Problems (ICD)-10 codes: S02, S12, S22, S32, S42, S52, S62, S72, S82, S92, T02, T08, T10, and T12) between 9 May 2007 (the first ever prescription of a GLP-1 RA in Denmark) and 31 December 2011. Controls were all subjects, both genders and aged 18 years and above, who did not sustain a fracture during the study period. We randomly selected one control for each case, matched by gender and year of birth. The controls were selected using incidence density sampling [16]. The date of the first fracture was used as the index date for cases, and controls were assigned the index date of their matched case.

Fractures were classified into the following categories using ICD-10 codes: hip (S72.0–S72.2), radius/ulna (S52), and vertebrae (S12, S22.0–S22.1, S32.0–S32.2, S32.7, S32.8, T08). A major osteoporotic fracture was defined as a fracture of the hip, radius/ulna, vertebrae, or humerus (S42.2–S42.4) according to the WHO definition [17].

### Exposure of Interest

We identified all drugs bought during the observation period using the Register of Medicinal Product Statistics. The dose of the drug was expressed as defined daily dose (DDD) [18]. ATC code A10B was used to determine exposure to non-insulin anti-diabetic drugs (NIAD), and patients were classified as current (1–91 days) or past (over 91 days) NIAD users, based on the time of the most recent prescription before the index date. Current NIAD users were divided into two mutually exclusive categories: never incretin users and incretin users.

To control for the potential that diabetes might act as a confounder [2], we employed never incretin use (i.e., current NIAD use excluding incretin use) as the reference category in our analysis. ATC codes A10BX04 and A10BX07 were used to determine GLP-1 RA exposure before the index date in the prescription database. Based on the time since the most recent prescription, cases and controls were classified as current (1–91 days), recent (92–182), or past (over 182 days) GLP-1 RA users. The DDDs estimated the cumulative dose of GLP-1 RAs for current GLP-1 RA users, expressed as exenatide equivalents. The average daily dose was estimated by dividing the cumulative exposure by the treatment time (time between the first GLP-1 RA prescription and the index date).

### Potential Confounders

A history of the following potential confounders ever before the index date were taken into account: chronic obstructive pulmonary disease (COPD), previous fracture, rheumatoid arthritis, hypothyroidism, hyperthyroidism, cancer, retinopathy, alcoholism, secondary osteoporosis (diabetes type 1, hypogonadism or premature menopause), and congestive heart failure. The potential confounders were identified from the National Hospital register using ICD-10 and ICD-8 codes. Additional potential confounders included a prescription in the 6 months before the index date of the following drugs: dipeptidyl peptidase 4 inhibitors (DPP4-Is), oral glucocorticoids [19], lipid-modifying drugs, antidepressants [20], anxiolytics, hypnotics [21], antipsychotics, anti-Parkinson drugs [22], antihypertensives [beta-blockers, thiazide diuretics, renin–angiotensin–aldosterone system (RAAS) inhibitors, calcium channel blockers, and loop diuretics], and antiarrhythmics. The prescription database was used to explore the presence of a prescription of the above-mentioned drugs.

### Statistical Analysis

Conditional logistic regression estimated the association between the use of GLP-1 RAs versus use of other anti-hyperglycemic drugs and risk of fracture, using the SAS 9.3 software. Analyses were stratified by age, gender, type of fracture, and for current GLP-1 RA use also by average daily dose and cumulative exposure. The stratified analyses were determined a priori, therefore no interaction analyses was performed. As a sensitivity analysis, we adjusted for current metformin use, as it has been associated with a decreased risk of fracture [6]. As a second sensitivity analyses, we adjusted the final model for insulin use, as insulin use has been associated with an increased fracture risk [5]. Final regression models were determined by stepwise backward elimination using a significance level of 0.05. All results are presented as odds ratios (OR) with the corresponding 95 % confidence intervals (CI).

## Results

### Study Population

The study population consisted of 229,145 cases and the same number of controls. The mean age was 55 years, and 56 % were women. Baseline characteristics are shown in Table 1.

**Table 1:** Baseline characteristics

Characteristic	Cases (*n* = 229,145)	Controls (*n* = 229,145)
Women	127,449 (55.6)	127,449 (55.6)
Mean age at index date (years, SD)	55 (20.6)	55 (20.6)
18–49 years	90,598 (39.5)	90,607 (39.5)
50–59 years	37,247 (16.3)	37,191 (16.2)
60–69 years	38,751 (16.9)	38,805 (16.9)
70–79 years	28,950 (12.6)	28,931 (12.6)
80+ years	33,599 (14.7)	33,611 (14.7)
History of comorbidities		
Type 1 diabetes	2113 (0.9)	1419 (0.6)
Alcoholism	11,147 (4.9)	4824 (2.1)
Fracture	46,446 (20.2)	15,418 (6.7)
Hyperthyroidism	3715 (1.6)	3688 (1.6)
Hypothyroidism	2887 (1.3)	2496 (1.1)
COPD	10,812 (4.7)	7,418 (3.2)
Congestive heart failure	7141 (3.1)	5424 (2.4)
Cancer	21,893 (9.6)	18,486 (8.1)
Rheumatoid arthritis	3912 (1.7)	2795 (1.2)
Retinopathy	3105 (1.4)	2314 (1.0)
Neuropathy	7915 (3.5)	6093 (2.7)
Secondary osteoporosis	5284 (2.3)	3352 (1.5)
Drug use within 6 months before index date		
Anti-hyperglycemic drugs	8541 (3.7)	8676 (3.8)
Biguanides	6223 (2.7)	6678 (2.9)
Sulfonylurea derivatives	3900 (1.7)	3809 (1.7)
Thiazolidinediones	262 (0.1)	183 (0.1)
Glinides	87 (0.0)	83 (0.0)
GLP-1 RAs	255 (0.1)	220 (0.1)
DPP4-Is	643 (0.3)	707 (0.3)
Insulins	4900 (2.1)	3261 (1.4)
Short acting	2046 (0.9)	1139 (0.5)
Intermediate acting	2049 (0.9)	1411 (0.6)
Long acting	1444 (0.6)	869 (0.4)
Combinations	1679 (0.7)	1200 (0.5)
Statins	31,874 (13.9)	32,064 (14.0)
Antiarrhythmics	818 (0.4)	522 (0.2)
Beta-blockers	23,281 (10.2)	23,592 (10.3)
Thiazide diuretics	20,425 (8.9)	21,547 (9.4)
RAAS inhibitors	37,555 (16.4)	39,379 (17.2)
Calcium channel blockers	22,942 (10.0)	22,816 (10.0)
Loop diuretics	16,905 (7.4)	12,766 (5.6)
Antidepressants	33,644 (14.7)	20,338 (8.9)
Anti-Parkinson drugs	3174 (1.4)	1814 (0.8)
Antipsychotics	8032 (3.5)	4867 (2.1)
Anxiolytics	14,668 (6.4)	10,431 (4.6)
Hypnotics	19,137 (8.4)	14,332 (6.3)
Glucocorticoids	9390 (4.1)	6858 (3.0)
Bisphosphonates	7371 (3.2)	4913 (2.1)
Raloxifene	214 (0.1)	148 (0.1)
Vitamin D	211 (0.1)	169 (0.1)
Calcium	1885 (0.8)	1375 (0.6)
Strontium ranelate	194 (0.1)	101 (0.0)
PTH	130 (0.1)	70 (0.0)
Calcitonin	2 (0.0)	1 (0.0)
Hormone replacement therapy	11,912 (5.2)	13,955 (6.1)
Beta2-agonists	10,436 (4.6)	8130 (3.6)
Inhaled anticholinergics	4569 (2.0)	3277 (1.4)
Inhaled corticosteroids	5265 (2.3)	4657 (2.0)

Data are number (%) of patients, unless stated otherwise

*SD* standard deviation, *GLP-1 RA* glucagon-like peptide-1 receptor agonist, *DPP4-I* dipeptidyl peptidase 4 inhibitor, *COPD* chronic obstructive pulmonary disease, *RAAS* renin angiotensin aldosterone system, *PTH* parathyroid hormone

Among cases, we identified 6993 (3.1 %) current NIAD users (excluding incretin users) and 255 (0.1 %) GLP-1 RA users (current, recent, or past). Similarly, we identified 7209 (3.1 %) NIAD users (excluding incretin users), and 220 (0.1 %) GLP-1 RA users among controls. The mean duration of actual GLP-1 RA use (from first GLP-1 RA prescription till index date) was 36 weeks.

### GLP-1 RA Use and Risk of Any Fracture

Current GLP-1 RA was not associated with a decrease in fracture risk: adjusted (adj.) OR 1.16, 95 % CI 0.83–1.63. Similarly, no significant decrease was observed for recent use (adj. OR 1.03, 95 % CI 0.69–1.53), or for past use (adj. OR 1.09, 95 % CI 0.82–1.46). Having no prior use of NIAD (never use) was associated with a significant increase in risk of fracture (adj. OR 1.10, 95 % CI 1.06–1.14), as well as past NIAD use (adj. OR 1.12, 95 % CI 1.05–1.20). The risk of fracture was not reduced after stratification by sex or age (Table 2).

**Table 2:** Use of GLP-1 RAs and risk of any fracture

Exposure	No. of cases (*N* = 229,145)^a^	No. of controls (*N* = 229,145)^a^	Crude OR (95 % CI)	Adjusted OR (95 % CI)^b^
Never NIAD use	217,623	218,194	1.03 (0.99–1.06)	1.10 (1.06–1.14)*
Past NIAD use	3631	2815	1.33 (1.25–1.41)*	1.12 (1.05–1.20)*
Current NIAD use excluding incretin use	6993	7209	Reference	Reference
Past GLP-1 RA use (183–365 days before index date)	120	93	1.33 (1.01–1.74)*	1.09 (0.82–1.46)
Recent GLP-1 RA use (92–182 days before the index date)	55	56	1.01 (0.70–1.47)	1.03 (0.69–1.53)
Current GLP-1 RA use (1–91 days before the index date)	80	71	1.16 (0.84–1.60)	1.16 (0.83–1.63)
By sex				
Males	38	39	0.98 (0.63–1.54)	0.97 (0.60–1.57)
Females	42	32	1.37 (0.86–2.18)	1.43 (0.87–2.32)
By age on index date				
<50 years	12	10	1.28 (0.52–3.11)	1.19 (0.47–2.98)
50–59 years	20	22	0.95 (0.51–1.75)	0.99 (0.51–1.89)
60–69 years	33	26	1.09 (0.64–1.86)	1.26 (0.71–2.24)
70+ years	15	13	1.29 (0.60–2.77)	1.04 (0.46–2.35)

Never NIAD use: no NIAD prescription before the index date

Past NIAD use: most recent NIAD prescription over 91 days before index date

Current NIAD use: most recent NIAD prescription within 91 days before index date

*OR* odds ratio, *CI* confidence interval, *GLP-1 RA* glucagon-like peptide-1 receptor agonist, *DPP4-I* dipeptidyl peptidase 4 inhibitor, *NIAD* non-insulin anti-diabetic-drugs, *COPD* chronic obstructive pulmonary disease, *RAAS* renin–angiotensin–aldosterone system

* Statistically significant, (*P* < 0.05)

^a^The numbers do not add up precisely to the total number of fractures because DPP4-I exposure is not shown

^b^Adjusted for history of cancer, COPD, fracture, alcoholism, rheumatoid arthritis, secondary osteoporosis, hyperthyroidism, retinopathy, neuropathy, heart failure and use of DPP4-Is, glucocorticoids, statins, anxiolytics, hypnotics, antidepressants, antipsychotics, anti-Parkinson drugs, beta-blockers, thiazide diuretics, RAAS inhibitors, loop diuretics, and antiarrhythmics

### Other Fracture Types

Table 3 shows that current GLP-1 RA use was not associated with osteoporotic fracture risk (adj. OR 0.78, 95 % CI 0.44–1.39). We identified similar trends for recent GLP-1 RA use (adj. OR 0.97, 95 % CI 0.52–1.79) and for past use (adj. OR 0.94, 95 % CI 0.60–1.48). Stratification by sex and age did not substantially change the results (data not shown). There was no significant association with fracture risk of the radius/ulna for current GLP-1 RA use (adj. OR 0.93, 95 % CI 0.48–1.80), recent GLP-1 RA use (adj. OR 1.32, 95 % CI 0.55–3.17), or for past GLP-1 RA use (adj. OR 0.93, 95 % CI 0.48–1.80). Stratification by gender and age did not show a decreased fracture risk (data not shown). Hip and vertebral fracture risk was not associated with GLP-1 RA use (current, recent, or past), Table 3.

**Table 3:** Use of GLP-1 RAs and fracture risk at different skeletal sites

Fracture sites	No. of cases^a^	No. of controls^a^	Crude OR (95 % CI)	Adjusted OR (95 % CI)
Osteoporotic fracture	96,774	96,774	–	–
Never NIAD use	90,297	90,677	1.02 (0.97–1.07)	1.07^b^ (1.02–1.13)*
Current NIAD use excl. incretin use	3957	4058	Reference	Reference
Past GLP-1 RA use (>182 days before the index date)	47	41	1.17 (0.77–1.78)	0.94^b^ (0.60–1.48)
Recent GLP-1 RA use (92–182 before the index date)	23	24	0.98 (0.55–1.73)	0.97^b^ (0.52–1.79)
Current GLP-1 RA use (0–91 days before the index date)	23	31	0.76 (0.44–1.30)	0.78^b^ (0.44–1.39)
Hip	24,328	24,328	–	–
Never NIAD use	21,923	22,273	0.94 (0.87–1.01)	1.00^c^ (0.92–1.09)
Current NIAD use excl. incretin use	1454	1388	Reference	Reference
Past GLP-1 RA use (>182 days before the index date)	9	4	2.11 (0.65–6.86)	1.74^c^ (0.47–6.49)
Recent GLP-1 RA use (92–182 before the index date)	5	4	1.18 (0.32–4.40)	1.34^c^ (0.26–6.93)
Current GLP-1 RA use (0–91 days before the index date)	4	4	0.99 (0.25–3.97)	0.77^c^ (0.16–3.73)
Radius/ulna	47,905	47,905	–	–
Never NIAD use	45,848	45,423	1.29 (1.19–1.39)*	1.29^d^ (1.19–1.40)*
Current NIAD use excl. incretin use	1273	1617	Reference	Reference
Past GLP-1 RA use (>182 days before the index date)	19	21	1.16 (0.62–2.16)	0.93^d^ (0.48–1.80)
Recent GLP-1 RA use (92–182 before the index date)	11	12	1.18 (0.52–2.68)	1.32^d^ (0.55–3.17)
Current GLP-1 RA use (0–91 days before the index date)	11	20	0.70 (0.34–1.47)	0.93^d^ (0.48–1.80)
Vertebral	9004	9004	–	–
Never NIAD use	8396	8450	1.01 (0.87–1.17)	1.11^e^ (0.93–1.32)
Current NIAD use excl. incretin use	364	369	Reference	Reference
Past GLP-1 RA use (>182 days before the index date)	4	5	0.80 (0.21–3.02)	0.97^e^ (0.22–4.32)
Recent GLP-1 RA use (92–182 before the index date)	2	4	0.50 (0.09–2.75)	0.30^e^ (0.05–1.84)
Current GLP-1 RA use (0–91 days before the index date)	2	2	1.00 (0.14–7.15)	2.02^e^ (0.28–14.75)

Never NIAD use: no NIAD prescription before the index date

Current NIAD use: most recent NIAD prescription within 91 days before index date

*OR* odds ratio, *CI* confidence interval, *GLP-1 RA* glucagon-like peptide-1 receptor agonist, *DPP4-I* dipeptidyl peptidase 4 inhibitor, *NIAD* non-insulin anti-diabetic-drugs, *COPD* chronic obstructive pulmonary disease, *RAAS* renin–angiotensin–aldosterone system

* Statistically significant, (*P* < 0.05)

^a^The numbers do not add up precisely to the total number of fractures because past NIAD use and DPP4-I exposure are not shown

^b^Adjusted for (f) and history of retinopathy, heart failure, COPD and use of glucocorticoids, statins, anxiolytics, antipsychotics, beta-blockers, thiazide diuretics, RAAS inhibitors, and antiarrhythmics

^c^Adjusted for (f) and history of retinopathy, heart failure, COPD and use of statins, anxiolytics, RAAS inhibitors, antiarrhythmics, glucocorticoids, and antipsychotics

^d^Adjusted for (f) and use of beta-blockers, thiazide diuretics, RAAS inhibitors, and calcium channel inhibitors

^e^Adjusted for (f) and history of COPD and use of glucocorticoids, anxiolytics, RAAS inhibitors and antipsychotics

^f^History of cancer, alcoholism, fracture, rheumatoid arthritis and secondary osteoporosis and use of DPP4-Is, antidepressants, anti-Parkinson drugs, loop diuretics, and hypnotics

### Current GLP-1 RA Use Stratified by Cumulative and Average Daily Dose and Fracture Risk

The risk of any fracture was comparable, and not significantly reduced, among the cumulative and average daily dose groups, Table 4. The adjusted OR for the highest cumulative dose (≥5.5 mg exenatide equivalent) was 0.98 (95 % CI 0.50–1.94) and the adjusted OR for the highest average daily dose (≥22.5 mcg exenatide equivalent per day) was 1.66 (95 % CI 0.86–3.19). When only osteoporotic fractures were considered, there was again no association with a decreased risk of fracture. Adjusted OR for the highest cumulative dose (≥5.5 mg exenatide equivalent) was 0.93 (95 % CI 0.30–2.92) and the adjusted OR for the highest average daily dose (≥22 mcg exenatide equivalent per day) was 1.40 (95 % CI 0.47–4.13).

**Table 4:** Use of GLP-1 RAs and risk of any fracture stratified by cumulative and average daily dose

	Any fracture			Osteoporotic fracture		
	No. of cases (*N* = 229,145)^a^	No. of controls (*N* = 229,145)^a^	Adjusted OR (95 % CI)^b^	No. of cases (*N* = 96,774)^a^	No. of controls (*N* = 96,774)^a^	Adjusted OR (95 % CI)^c^
Current NIAD use excl. incretins	6993	7209	Reference	3957	4058	Reference
Current GLP-1 RA use	80	71	1.16 (0.83–1.63)	23	31	0.78 (0.44–1.39)
By cumulative exposure^d^						
0–1.4 mg	36	34	1.05 (0.63–1.73)	10	17	0.64 (0.28–1.47)
1.4–2.7 mg	14	11	1.60 (0.69–3.69)	3	5	0.57 (0.12–2.74)
2.7–5.5 mg	12	8	1.55 (0.60–3.95)	4	2	2.05 (0.32–13.30)
≥5.5 mg	18	18	0.98 (0.50–1.94)	6	7	0.93 (0.30–2.92)
By average daily dose^d^						
<15 mcg/day	16	19	0.91 (0.47–1.84)	3	9	0.35 (0.09–1.39)
15–22.49 mcg/day	38	36	1.07 (0.66–1.73)	12	15	0.78 (0.35–1.75)
≥22.5 mcg/day	26	16	1.66 (0.86–3.19)	8	7	1.40 (0.47–4.13)
Recent GLP-1 RA use	55	56	1.03 (0.69–1.53)	23	24	0.97 (0.52–1.79)
Past GLP-1 RA use	120	93	1.09 (0.82–1.46)	47	41	0.94 (0.60–1.48)

*OR* odds ratio, *CI* confidence interval, *GLP-1 RA* glucagon-like peptide-1 receptor agonist, *DPP4-I* dipeptidyl peptidase 4 inhibitor, *NIAD* non-insulin anti-diabetic-drugs, *COPD* chronic obstructive pulmonary disease, *RAAS* renin–angiotensin–aldosterone system

* Statistically significant, (*P* < 0.05)

^a^The numbers do not add up precisely to the total number of fractures because never NIAD, past NIAD and DPP4-I use are not shown

^b^Adjusted for history of cancer, alcoholism, COPD, fracture, rheumatoid arthritis, hyperthyroidism, secondary osteoporosis, retinopathy, neuropathy and heart failure and use of DPP4-Is, glucocorticoids, statins, antidepressants, anxiolytics, hypnotics, antipsychotics, anti-Parkinson drugs, beta-blockers, thiazide diuretics, RAAS inhibitors, loop diuretics, antiarrhythmics

^c^Adjusted for (a), but not for hyperthyroidism and neuropathy

^d^In exenatide equivalents

### Sensitivity Analysis

Adjusting the main analysis for current metformin use did not substantially change our results. The adjusted OR for current GLP-1 RA use was 1.17 (95 % CI 0.83–1.64), for recent GLP-1 RA 1.03 (95 % CI 0.70–1.54), and for past GLP-1 RA use 1.09 (95 % CI 0.82–1.46).

As a second sensitivity analysis, we additionally adjusted the main analysis for insulin use and this did not alter the results. The adjusted OR for current GLP-1 RA use was 1.13 (0.80–1.58), for recent use 1.00 (0.67–1.49), and for past use 1.05 (0.79–1.41).

## Discussion

The present study showed that current GLP-1 RA use was not associated with a decreased risk of any fracture, as compared to the use of other anti-hyperglycemic drugs, and current GLP-1 RA use was not associated with a reduced risk of other fracture types. Moreover, stratification of current GLP-1 RA by cumulative or average daily dose was not associated with a decreased risk of fracture.

The results of the present study are in line with the results of a meta-analysis of clinical trials on the effect of GLP-1 RAs on fracture risk, which showed that fracture risk was not significantly reduced with use of GLP-1 RA [11, 23]. Our results are also in keeping with a large clinical trial (*n* = 16,492) on the effect of a DPP4-I, saxagliptin, which showed no difference in risk of fracture with the use of DPP4-I use and placebo [24]. Additionally, the present results are also supported by the results of a cohort study which was not able to show a decreased risk of fracture with the use of GLP-RA as compared to the use of other anti-hyperglycemic drugs [12] and a cohort study that compared the use of DPP4-I to the use of other anti-hyperglycemic drugs [25]. The pathway by which DPP4-Is might affect bone metabolism may be the same as that of GLP-1 RA because DPP4-Is inhibits the degradation of GLP-1 [2]. Moreover, our results are indirectly supported by a clinical trial on the effect of a GLP-1 RA, exenatide, on markers of bone metabolism [26], which reported that bone markers were unaffected after 44 weeks of exenatide treatment.

We acknowledge that our findings are in contrast to those of a meta-analysis of randomized clinical trials (*n* = 22,055) that showed a significant 40 % reduction of fracture risk with the use of DPP4-Is [27] as compared to active treatment or placebo. However, studies that were included in this meta-analysis did not routinely collect fractures as an outcome of interest, the number of fractures was low, and different comparator groups had been used [27]. The results from this meta-analysis should likely be interpreted with caution as the low number of events in a meta-analysis of adverse events can give biased estimates [28, 29].

Results of in vitro studies have suggested that the use of GLP-1 RAs might have a beneficial effect on bone metabolism [7–9], yet different mechanisms of effect have been hypothesized. In vitro studies have shown that GLP-1 receptors are present on bone marrow stromal cells [30], immature osteoblast [31] and osteocytes [10], and binding of GLP-1 to its receptor on bone cells leads to increased osteoblast activity [9], and decreased osteoclast activity [10]. This could then lead to increased bone formation and a reduced fracture risk. However, more research is needed to assess whether GLP-1 RAs are also able to bind to GLP-1 receptors on human bone cells and whether this could ultimately result in a reduced risk of fracture.

An unexpected finding was that never and past NIAD use showed an increased fracture risk as compared to the current use of other anti-hyperglycemic drugs. This may have been the result of residual confounding by high body mass index (BMI), which has been shown to decrease fracture risk [32]. T2DM has been associated with an increased BMI and therefore it might be that the increased risk of never NIAD use is a result of a higher BMI in the current NIAD use group which was used as a reference group. The past NIAD use group included patients with T2DM who use insulin only. Insulin use has been associated with a twofold increased risk of fracture as compared to patients with T2DM who use NIADs [2].

There are several strengths to the current study. First the use of a nation-wide population register permitted the examination of a large number of cases and controls. The data used were also collected longitudinally, and for prescriptions this permitted us to calculate a reliable cumulative and average daily dose. Second, the data used to identify fractures have been validated [13]. Third, we were able to adjust our analyses for many potential confounders. When interpreting our results, we are mindful of a couple of limitations. We were not able to adjust for potential confounders such as BMI, hemoglobin A1c (HbA1c), smoking, amount of exercise, and serum vitamin D levels. A high BMI has been associated with a reduced risk of fracture [2] and therefore a decreased risk of fracture with GLP-1 RA use could have represented the association between high BMI and fracture risk. Nevertheless, we did not show a reduced fracture risk with GLP-1 RA use. Smoking is a risk factor of fracture [33], but there is no evidence that GLP-1 RA users have different smoking behaviors than the patients treated with other anti-hyperglycemic drugs. Exercise has been associated with a decreased risk of fracture [34]; GLP-1 RA users might perform less exercise which could result in an overestimation of the effect. Lower levels of serum vitamin D have been associated with more severe T2DM complications and increased fracture risk [35, 36]. Not adjusting for serum vitamin D levels might lead to an overestimation of the effect when GLP-1 RA users have lower serum vitamin D levels as compared to the users of other anti-hyperglycemic drugs. Thus, while we were able to identify a large number of confounders due to the completeness of the registry data, it is acknowledged that some residual confounding might still be present. In addition, we tried to capture severity of diabetes by correcting our analyses for known complications of diabetes, such as neuropathy and retinopathy. Moreover, we used current NIAD use (excluding incretin use) as a reference group, because diabetes itself might act as a confounder. Current NIAD use included use of TZDs or insulins, which have been shown to increase fracture risk [3, 4]. The result of this bias in the reference category may be an observed artificial inverse association between GLP-1 RAs use and risk of fracture which could have falsely supported our hypothesis. However, we did not observe an inverse association between GLP-1 RA use and risk of fracture. Current NIAD use also included metformin use which has been associated with a reduced fracture risk and therefore it might mask a decreased association between GLP-1 RA use and fracture risk. Nevertheless, statistical adjustment of the main analysis for current metformin use did not alter the results.

Although the total number of fractures was high, the number of some fracture types (i.e., hip and radius/ulna) was not high enough to stratify current GLP-1 RA use by cumulative and average dose, and to keep adequate statistical power. In the analyses, with hip and vertebral fracture as outcome, the number of GLP-1 RA users was quite small, therefore these results should be interpreted with caution. The average duration of actual GLP-1 RA use (36 weeks) in the present study was rather short, which might be the reason that we were unable to observe an association between GLP-1 RA use and fracture risk. However, after stratification of current GLP-1 RA use by cumulative dose, risk of fracture was not decreased in the group with patients who had used on average 15 microgram exenatide equivalent per day for at least 1 year (cumulative dose: ≥5.5 mg exenatide equivalent).

In conclusion, we showed in a population-based case–control study that the use of GLP-1 RAs (current, recent or past) is not associated with fracture risk as compared to the use of other anti-hyperglycemic drugs. In addition, current GLP-1 RA use stratified by cumulative and average daily dose was not associated with a decreased fracture risk. More research is needed, and in particular future studies should focus on the effect of long-term use of GLP-1 RAs on fracture risk.
